# Zinc finger and SCAN domain-containing protein 18 is a potential DNA methylation-modified tumor suppressor and biomarker in breast cancer

**DOI:** 10.3389/fendo.2023.1095604

**Published:** 2023-05-08

**Authors:** Yu Wang, Yuhao Luo, Shaozhi Fu, Lijia He, Guangrui Pan, Dongmei Fan, Qinglian Wen, Yu Fan

**Affiliations:** ^1^ Health Management Department, The Affiliated Hospital of Southwest Medical University, Luzhou, China; ^2^ Department of Oncology, The Affiliated Hospital of Southwest Medical University, Nuclear Medicine and Molecular Imaging Key Laboratory of Sichuan Province, Academician (Expert) Workstation of Sichuan Province, Luzhou, China; ^3^ Department of Breast Surgery, The Affiliated Hospital of Southwest Medical University, Luzhou, China; ^4^ Department of Nuclear Medicine, The Affiliated Hospital of Southwest Medical University, Luzhou, China

**Keywords:** DNA methylation, ZSCAN18, breast cancer, transcription regulation, tumor suppressor, integrated analysis

## Abstract

**Introduction:**

Zinc finger and SCAN domain-containing protein 18 (ZSCAN18) has been investigated as a putative biomarker of multiple human cancers. However, the expression profile, epigenetic modification, prognostic value, transcription regulation, and molecular mechanism of ZSCAN18 in breast cancer (BC) remain unknown.

**Methods:**

In the study, we present an integrated analysis of ZSCAN18 in BC based on public omics datasets with the use of multiple bioinformatics tools. Genes potentially regulated through restoration of ZSCAN18 expression in MDA-MB-231 cells were investigated to identify pathways associated with BC.

**Results:**

We observed that ZSCAN18 was downregulated in BC and mRNA expression was significantly correlated with clinicopathological parameters. Low expression of ZSCAN18 was found in the HER2-positive and TNBC subtypes. High expression of ZSCAN18 was associated with good prognosis. As compared to normal tissues, the extent of ZSCAN18 DNA methylation was greater with fewer genetic alterations in BC tissues. ZSCAN18 was identified as a transcription factor that might be involved in intracellular molecular and metabolic processes. Low ZSCAN18 expression was associated with the cell cycle and glycolysis signaling pathway. Overexpression of ZSCAN18 inhibited mRNA expression of genes associated with the Wnt/β-catenin and glycolysis signaling pathways, including CTNNB1, BCL9, TSC1, and PFKP. ZSCAN18 expression was negatively correlated with infiltrating B cells and dendritic cells (DCs), as determined by the TIMER web server and reference to the TISIDB. ZSCAN18 DNA methylation was positively correlated with activated B cells, activated CD8+ and CD4+ T cells, macrophages, neutrophils, and activated DCs. Moreover, five ZSCAN18-related hub genes (KDM6B, KAT6A, KMT2D, KDM1A, and HSPBP1) were identified. ZSCAN18, ZNF396, and PGBD1 were identified as components of a physical complex.

**Conclusion:**

ZSCAN18 is a potential tumor suppressor in BC, as expression is modified by DNA methylation and associated with patient survival. In addition, ZSCAN18 plays important roles in transcription regulation, the glycolysis signaling pathway, and the tumor immune microenvironment.

## Introduction

Breast cancer (BC) is the second leading cause of cancer-related death in women worldwide (1, 2). Although tumor suppressor genes normally act to inhibit cell proliferation and tumor development, deletion and insertion mutations lead to frame shifts in the DNA nucleotide sequence that can promote the development of BC (3). DNA methylation is a common event in the early development of various tumors. Therefore, targeting of DNA methylation sites provides a new strategy for the treatment of BC.

As the largest family of transcription factors (TFs) in the human genome, zinc finger (ZNF) proteins play important roles in many biological processes (4). The structure of ZNF proteins is stabilized by one or more zinc ions, while transcription is regulated through interactions of N-terminal domains with other proteins (5). Zinc finger and SCAN domain-containing (ZSCAN) TFs constitute the smallest and most recently defined subfamily of ZNF proteins (6). Numerous studies have reported abnormal expression of ZSCAN TFs in various malignant tumors. Notably, the activities of ZSCAN TFs often vary among different tumor types and even within the same tumor type. Recent studies have implicated ZSCAN TFs in angiogenesis, apoptosis, cell differentiation, proliferation, migration, and invasion, stem cell characteristics, and sensitivity to chemotherapeutic drugs (7). Mutations, DNA methylation status, alternative splicing events, and miRNA activities influence the regulation of ZSCAN TFs. ZSCAN18 methylation is prevalent and negatively correlated with expression in multiple human cancers, and the potential of ZSCAN18 methylation as a marker for tumor diagnosis and prognosis has been discovered. ZSCAN18 expression can be reversed in a variety of renal cancer cell lines after treatment with demethylated drug. In a recent study, inactivation of ZSCAN18 by DNA methylation drives the proliferation *via* attenuating TP53INP2-mediated autophagy in gastric cancer. Therefore, previous studies have linked ZSCAN18 to the development of various human cancers (8–14). However, the role of ZSCAN18 in BC remains unknown.

Therefore, the aim of this study was to clarify the expression profile of ZSCAN18 and the mechanisms underlying epigenetic regulation in BC as a potential prognostic biomarker.

## Materials and methods

### Expression profile analysis

The mRNA expression of ZSCAN18 was analyzed by tumor immune estimation resource (TIMER), the University of Alabama at Birmingham cancer data analysis portal (UALCAN), and breast cancer gene-expression miner (bc-GenExMiner). The mRNA expression profile of ZSCAN18 in multiple human cancers was investigated by the DiffExp module of TIMER (https://cistrome.shinyapps.io/timer/), which study the differential expression between tumor and adjacent normal tissues across all the cancer genome atlas (TCGA) tumors (15). The ZSCAN18 mRNA expression for primary tumor (1,097 cases) and normal tissues (114 cases) was downloaded from UALCAN (http://ualcan.path.uab.edu/) (16), which based on TCGA for breast invasive carcinoma (TCGA-BRCA). Moreover, bc-GenExMiner (http://breastcancergenex.ico.unicancer.fr/) (17) was used to assess the mRNA expression of ZSCAN18 in healthy, tumor-adjacent, and tumor cases, based on TCGA and GTEx dataset. The relationships among ZSCAN18 expression and clinicopathological parameters including age, hormone receptor (estrogen receptor (ER) and progesterone receptor (PR)), human epidermal growth factor receptor type 2 (HER2), basel-like, triple negative breast cancer (TNBC), Nottingham prognostic index (NPI), Scarff-Bloom-Richardson (SBR) grade, tumor stage, and P53 mutation, were also evaluated. The protein expression of ZSCAN18 in primary tumor of breast cancer and normal tissues was analyzed by UALCAN, based on clinical proteomic tumor analysis consortium (CPTAC) dataset. The expression level of ZSCAN18 in breast cancer cell lines was acquired through depmap portal of cancer cell line encyclopedia (CCLE; https://depmap.org/portal/), in which gene expression transcript per million (TPM) values are inferred from RNA-seq data using the RSEM tool and are reported after log2 transformation, using a pseudo-count of 1 (log2(TPM+1)) (18).

### DNA methylation and genetic alteration analysis

The genetic alteration and promoter methylation of ZSCAN18 in breast cancer were displayed through cBioPortal (https://www.cbioportal.org/) (METABRIC, Nature 2012 & Nat Commun 2016, 1904 samples) (19). To further access the expression regulatory effect of DNA methylation modification on ZSCAN18 mRNA, we used the EMBOSS cpgplot explorer (https://www.ebi.ac.uk/Tools/seqstats/emboss_cpgplot/) of European molecular biology laboratory’s European bioinformatics institute (EMBL-EBI) to identify and plot CpG islands in nucleotide sequence (20). The promoter methylation of ZSCAN18 in primary tumor and normal tissues was further analyzed based on TCGA-BRCA from UALCAN. We also used MEXPRESS (https://mexpress.be/) (21) to visualize DNA methylation, expression and clinical data of ZSCAN18 in breast invasive carcinoma.

### Prognostic value analysis

The Kaplan-Meier plotter (KM plotter; http://kmplot.com/analysis/) (22), is a web online tool capable to assess the correlation between the expression and survival from 21 tumor types including breast, ovarian, lung, and gastric cancer based on datasets including GEO, EGA, and TCGA, was used to predict the prognostic value of ZSCAN18 in breast cancer. The hazards ratio (HR), 95% confidence interval (CI), and log-rank p were displayed on the web page.

### Gene transcription regulation and gene ontology analysis

In this study, we used gene transcription regulation database (GTRD; http://gtrd.biouml.org/) (23), the most complete collection of uniformly processed ChIP-seq data on identification of transcription factor binding sites for human and mouse, to find potential genes regulated by ZSCAN18. Gene ontology (GO) and pathway enrichment analysis of ZSCAN18-regulated genes were performed using Metascape (http://metascape.org/) (24). The top clusters with their enriched terms, which originated from several categories, were included.

### Gene set enrichment analysis (GSEA)

A powerful analytical method called GSEA for interpreting gene expression data was used to evaluate whether a prior defined set of genes shows statistically significant (25). The gene sets are defined based on prior biological knowledge, published information about biochemical pathways or co-expression in previous experiments. An ordered list of genes was first generated based on association with ZSCAN18 expression. Gene set permutations were performed 1,000 times. The expression level of ZSCAN18 was used as phenotype label. The nominal p-value and normalized enrichment score (NES) were used to classify the Kyoto Encyclopedia of Genes and Genomes (KEGG) pathways enriched in low phenotype.

### Immune infiltration analysis

Gene module of TIMER allows users to select any gene of interest and visualize the correlation of its expression with immune infiltration level in diverse cancer types. The relationship of ZSCAN18 expression in breast invasive carcinoma with the abundance of immune infiltration, including B cells, CD8+ T cells, CD4+ T cells, macrophages, neutrophils, and dendritic cells (DCs), was analyzed by using gene module of TIMER. Gene expression level normalized with tumor purity was displayed on the left most panel.

Tumor-immune system interaction database (TISIDB; http://cis.hku.hk/TISIDB/) (26) is an online portal for tumor and immune system interaction, which can integrate relationships between abundance of tumor-infiltrating lymphocytes (TILs) and expression, methylation, copy number, or mutation of current gene. The immune-related signatures of 28 TIL types from Charoentong’s study, which can be viewed in the download page. For each cancer type, the relative abundance of TILs was inferred by using gene set variation analysis (GSVA) based on gene expression profile. In this tab, users can examine which kinds of TILs might be regulated by the current gene. The relationship of ZSCAN18 expression/methylation with abundance of TILs (including activated B cell, activated CD8 T cell, activated CD4 T cell, macrophage, neutrophil, and activated DCs) was investigated in TISIDB database.

### Hub genes Identification and protein-protein interaction (PPI)

The top five ZSCAN18-related hub genes were identified using the CytoHubba plugin of Cytoscape that used the degree method. The expression of hub genes and the associations with overall survival (OS) in breast cancer were evaluated by bc-GenExMiner and KM plotter. STRING was used to perform a PPI physical subnetwork of ZSCAN18 with an interaction score of >0.7.

### Quantitative reverse transcription polymerase chain reaction (qRT-PCR)

The EX-Z7012-Lv201 ZSCAN18 vectors were generated based on pEZ-Lv201 (Genecopoeia, Rockville, MD, USA). The lentivirus particles were produced by co-transfecting the corresponding vectors with packaging GV plasmids (Genechem, Shanghai, China) in 293T cells. Supernatants containing ZSCAN18 lentivirus were gathered and filtered to infect MDA-MB-231. Total RNA was extracted from MDA-MB-231 cells by Trizol. 2×Hifair^®^ II SuperMix plus (cat. no. 11123ES60, YEASEN, Shanghai, China) was used for reverse transcription, and Hieff^®^ qPCR SYBR Green Master Mix (Low Rox) (cat. no. 11202ES08, YEASEN, Shanghai, China) was utilized for the qRT-PCR according to the manufacturer’s protocol. The primer sequences are shown in **Table S1**. The thermocycling process was shown: initial denaturation for 10 min at 95°C, 40 cycles of denaturation for 15 s at 95°C, and 40 cycles of amplification for 1 min at 60°C. The mRNA expression of CTNNB1, BCL9, TSC, LDHA, and PFKP in cells was represented as the 2^−ΔΔCt^, and β-actin was the internal reference.

## Results

### Expression profile of ZSCAN18 in BC

**Figure 1:**
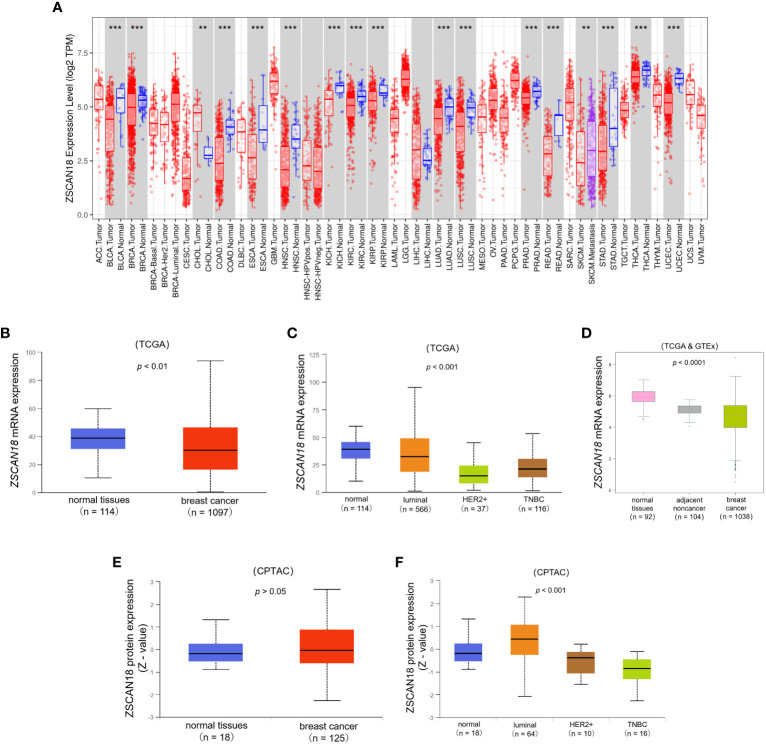
The expression profile of ZSCAN18 in BC. **(A)** The mRNA expression of ZSCAN18 in multiple human cancers by comparison of tumor and adjacent normal tissues based on TCGA datasets constructed by TIMER, which identifying genes that are upregulated or downregulated in the tumors compared to normal tissues for each cancer type, as displayed in gray columns when normal data are available; BRCA. Tumor, breast cancer; BRCA. Normal, breast normal tissues; **p < 0.01; ***p < 0.001; **(B)** The comparison of ZSCAN18 mRNA expression in breast primary tumor and normal tissues based on TCGA datasets constructed by UALCAN; **(C)** The mRNA expression of ZSCAN18 in tumor subtypes of breast invasive carcinoma; **(D)** The ZSCAN18 mRNA level in breast tumor, healthy, and tumor-adjacent tissues based on TCGA and GTEx datasets; **(E)** The protein expression of ZSCAN18 in primary tumor of BC and normal tissues and **(F)** in tumor subtypes of breast invasive carcinoma. Z-values represent standard deviations from the median across samples for the given cancer type. Log2 Spectral count ratio values from CPTAC were first normalized within each sample profile, and normalized across samples.

TCGA dataset was referenced to determine the mRNA expression profiles and potential roles of ZSCAN18 in multiple types of cancer. The TIMER web server was used to compare the mRNA expression patterns of ZSCAN18 in tumor and adjacent normal tissues. As shown in **Figure 1A**; **Table S2**, mRNA expression, calculated as Log2 TPM, of ZSCAN18 was downregulated in invasive BC tissues as compared to adjacent normal tissues (p < 0.001). Meanwhile, the TPM of ZSCAN18 was lower in primary tumor tissues (n = 1097) than normal tissues (n = 114) from TCGA samples, as determined with the UALCAN data analysis portal (p = 0.007, < 0.01) **(** **Figure 1B** **)**. Except in luminal subtype of BC, the TPM of ZSCAN18 in the HER2-positive and TNBC subtypes were both much lower than normal tissues (p < 0.001) **(** **Figure 1C** **)**. The statistical significance of ZSCAN18 mRNA expression among different tumor subtypes of invasive BC is shown in **Table S3**. The mRNA expression profile of ZSCAN18 was validated using the TCGA and GTEx datasets. As shown in **Figure 1D**, the Log2 standardized mRNA level of ZSCAN18 was lower in tumor tissues (n = 1038) than healthy and tumor-adjacent tissues (n = 92 and 104, respectively) (p < 0.0001). Moreover, ZSCAN18 mRNA expression profiles, calculated as Log2 (TPM+1), in BC cell lines were acquired from the CCLE **(** **Table S4** **)**. Notably, ZSCAN18 expression was relatively low in some BC cell lines.

At the protein level, there was no significant difference in ZSCAN18 expression between primary tumor and normal tissues (p = 0.606, > 0.05), but was much lower in the HER2-positive and TNBC subtypes than the luminal subtype of BC (p < 0.001) **(** **Figures 1E, F** **)**. The statistical significance of ZSCAN18 protein expression among different tumor subtypes of invasive BC is shown in **Table S5**. Collectively, these results demonstrate that ZSCAN18 expression is significantly downregulated in BC, especially in the subtypes of HER2-positive and TNBC.

**Figure 2:**
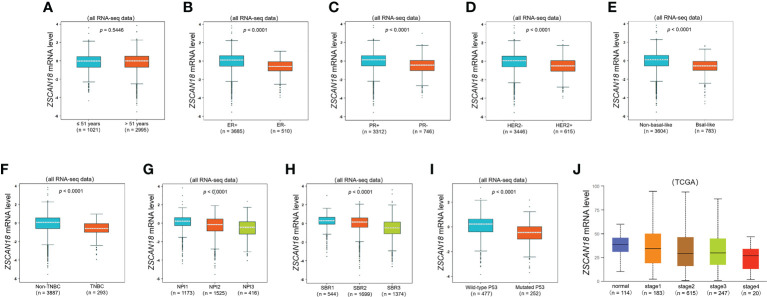
The relationships among ZSCAN18 expression and clinicopathological significance of BC constructed by bc-GenExMiner. **(A)** The mRNA level of ZSCAN18 in BC patients with different ages (≤ 51 and > 51); **(B)** The mRNA level of ZSCAN18 in BC patients with different ER status (ER-positive and ER-negative); **(C)** The mRNA level of ZSCAN18 in BC patients with different PR status (PR-positive and PR-negative); **(D)** The mRNA level of ZSCAN18 in BC patients with different HER2 status (HER2-negative and HER2-positive); **(E)** The mRNA level of ZSCAN18 in BC patients with different basal-like status (Non-basal-like and Basal-like); **(F)** The mRNA level of ZSCAN18 in BC patients with different TNBC status (Non-TNBC and TNBC); **(G)** The mRNA level of ZSCAN18 in BC patients with different NPI (NPI1, NPI2, and BPI3); **(H)** The mRNA level of ZSCAN18 in BC patients with different SBR (SBR1, SBR2, and SBR3); **(I)** The mRNA level of ZSCAN18 in BC patients with different P53 status (Wild-type and Mutated) and **(J)** the mRNA level of ZSCAN18 in BC patients with different tumor stages. The p value indicated statistical significance.

To further evaluate the potential role and clinical significance of ZSCAN18 in BC, the bc-GenExMiner mining tool was used to evaluate the associations of ZSCAN18 expression with clinicopathological parameters **(** **Figure 2** **)**. The results revealed no significant difference in ZSCAN18 expression between patients aged ≤51 vs. >51 years (p = 0.8714, >0.05). HER2 status, the basal-like and TNBC subtypes, tumor stage, NPI, SBR grade, and mutated tumor protein P53 were negatively associated with ZSCAN18 expression at the mRNA level (p < 0.0001). The statistical significance of ZSCAN18 TPM in the tumor stage of invasive BC is described in **Table S6**. Notably, ZSCAN18 mRNA expression was significantly upregulated in ER and PR-positive BC (p < 0.0001).

### Prognostic significance of ZSCAN18 in BC

**Figure 3:**
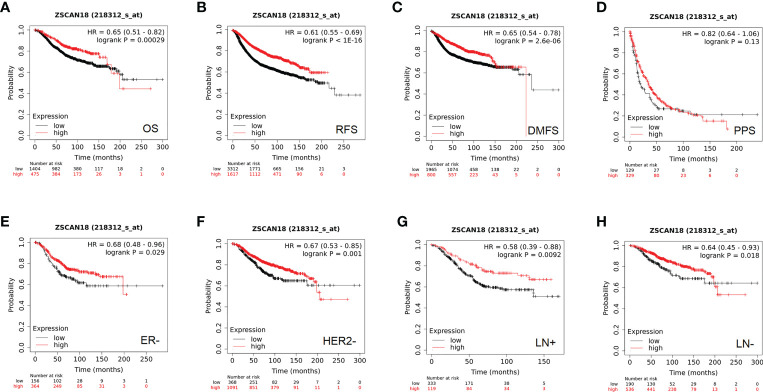
The prognostic significance of ZSCAN18 in BC. **(A)** High ZSCAN18 mRNA expression shows a better OS for BC; **(B)** High ZSCAN18 mRNA expression shows a better RFS for BC; **(C)** High ZSCAN18 mRNA expression shows a better DMFS for BC; **(D)** High ZSCAN18 mRNA expression shows a better PPS for BC; **(E)** High ZSCAN18 mRNA expression shows a better OS for ER negative (ER-) BC; **(F)** High ZSCAN18 mRNA expression shows a better OS for HER2 negative (HER2-) BC; **(G)** High ZSCAN18 mRNA expression shows a better OS for lymph node positive (LN+) BC; **(H)** High ZSCAN18 mRNA expression shows a better OS for lymph node negative (LN-) BC. HR, 95% CI, and log-rank p were displayed.

Based on the best cutoff value, analysis of 1,879 BC patients using the Kaplan–Meier plotter demonstrated that high mRNA expression of ZSCAN18 was significantly associated with improved OS (HR = 0.65; 95% CI = 0.51–0.82; p = 0.00029, < 0.001, **Figure 3A**) and relapse-free survival (RFS) in a cohort of 4,929 BC patients (HR = 0.61; 95% CI = 0.55–0.69; p < 1E-16, < 0.001, **Figure 3B**). Although there was no significant difference in post-progression survival (PPS) between the high and low expression groups, ZSCAN18 was positively correlated with distant metastasis-free survival (DMFS) in BC (HR = 0.65; 95% CI = 0.54–0.78; p = 2.6E-06, < 0.001, **Figures 3C, D**). Taken together, these results demonstrate the potential of ZSCAN18 as a prognostic biomarker for BC patients.

In addition, the prognostic value of ZSCAN18 in different molecular subtypes of BC and lymph node status was investigated using the Kaplan–Meier plotter. Although there was no remarkable difference in OS among the luminal A, luminal B, HER2-positive, basal-like, and ER-positive subtypes of BC **(** **Figure S1** **)**, high ZSCAN18 mRNA expression was correlated with better OS for the subtypes ER-negative (HR = 0.68; 95% CI = 0.48–0.96, p = 0.029, < 0.05), HER2-negative (HR = 0.67; 95% CI = 0.53–0.85, p = 0.001, < 0.01) lymph node-positive (HR = 0.58; 95% CI = 0.39–0.88, p = 0.0092, < 0.01), and lymph node-negative (HR = 0.64; 95% CI = 0.45–0.93, p = 0.018, < 0.05) **(** **Figures 3E–H** **)**.

### DNA methylation status and genetic alteration of ZSCAN18 in BC

**Figure 4:**
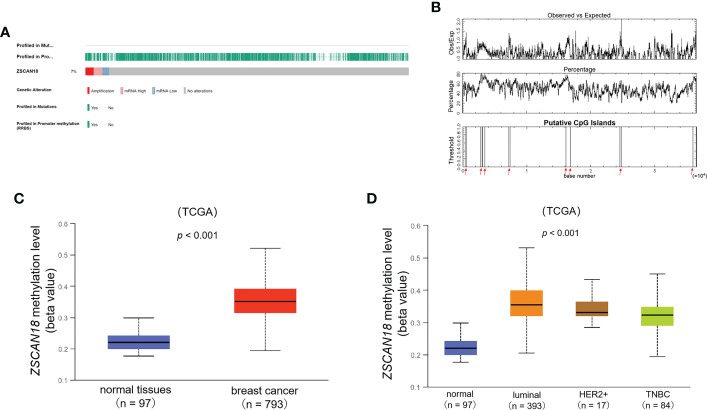
DNA methylation and genetic alteration of ZSCAN18 in BC. **(A)** Promoter methylation, gene mutation, and genetic alteration profile of ZSCAN18 are constructed by the cBioPortal; Tumor samples are shown in columns. General promoter methylation but no gene mutation is found in the ZSCAN18 nucleotide sequence; Genomic alterations of ZSCAN18 are mutually exclusive. **(B)** The “Putative CpG Islands” in nucleotide sequence of ZSCAN18, and which are marked by red arrow. “Observed vs Expected”, the minimum average observed to expected ratio of C plus G to CpG in a set of 10 windows are required before a CpG island is reported (default value: 0.6); “Percentage”, the minimum average percentage of G plus C in a set of 10 windows are required before a CpG island is reported (default value: 50). **(C)** The promoter methylation level of ZSCAN18 in primary tumor and normal tissues, in which the beta value indicates level of DNA methylation ranging from 0 (unmethylated) to 1 (fully methylated). Different beta value cut-off has been considered to indicate hypermethylation (beta value: 0.7 - 0.5) or hypomethylation (beta-value: 0.3 - 0.25); **(D)** The promoter methylation level of ZSCAN18 in tumor subtypes of breast invasive carcinoma and normal tissues. The p value indicated statistical significance.

Further analysis with the cBioPortal tool found general promoter methylation but no gene mutation to the ZSCAN18 nucleotide sequence. Genetic alterations to ZSCAN18 were detected in 141 (7%) of 1904 BC patients/samples, including 50 with DNA amplification **(** **Figure 4A** **)**. CpG islands in the nucleotide sequence of ZSCAN18 were identified and plotted using the cpgplot tool included with the EMBOSS explorer suite of bioinformatics tools. The results are presented in **Figure 4B**. The TCGA dataset from UALCAN was referenced to determine whether low expression of ZSCAN18 is associated with promoter methylation in BC. The results showed that the extent of methylation of the ZSCAN18 promoter was significantly higher in primary tumor tissues (n = 793) than normal tissues (n = 97) (p < 0.001) **(** **Figure 4C** **)**. Meanwhile, the extent of methylation the ZSCAN18 promoter was relatively higher for the luminal (n = 393), HER2-positive (n = 17), and TNBC (n = 84) subtypes of BC than normal tissues (n = 97) (p < 0.001) **(** **Figure 4D** **)**. The statistical significance of the extent of methylation of the ZSCAN18 promoter among different subtypes of invasive BC is described in **Table S7**. Furthermore, the MEXPRESS data visualization tool was employed to visualize DNA methylation and expression data of ZSCAN18 in invasive BC. Based on Pearson’s correlation coefficient, ZSCAN18 mRNA expression was negatively correlated with the extent of methylation of the CpG islands **(** **Figure S2** **)**. These results support the important role of DNA methylation in regulation of ZSCAN18 expression.

### Genes regulated by ZSCAN18 and related signaling pathways in BC

**Figure 5:**
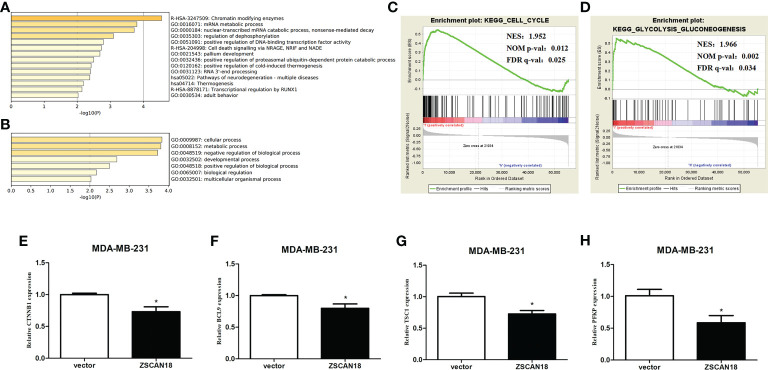
Genes transcription regulated by ZSCAN18 and its potential signaling pathways in BC. **(A)** The top 14 pathway enrichment clusters were found based on ZSCAN18 regulated genes and carried out with GO Biological Processes, KEGG pathway, and Reactome Gene Sets through Metascape. **(B)** The top-level GO biological processes were found based on ZSCAN18 regulated genes through Metascape. Length of bars represent log_10_ (p-value) determined by the best-scoring term within each cluster. Cell cycle **(C)** and glycolysis signaling pathway **(D)** differentially enriched in ZSCAN18-low expression phenotype constructed by GSEA. Gene sets with FDR q-val<0.05 are considered as significant. NOM p-value: normalized p-value; FDR q-value: false discovery rate q-value. **(E–H)** The mRNA expressions of CTNNB1, BCL9, TSC, and PFKP in ZSCAN18 overexpressed MDA-MB-231 cell line. *p < 0.05.

The GTRD was referenced to elucidate the potential role and underlying regulatory mechanism of ZSCAN18 in BC. The results identified 122 genes potentially regulated by ZSCAN18 **(** **Table S8** **)**. The Metascape gene annotation and analysis resource was used for pathway enrichment analysis and identification of biological processes. Terms with a p-value < 0.01, minimum count = 3, and enrichment factor (ratio between the observed counts and the counts expected by chance) > 1.5 were collected and grouped into clusters based on membership similarities. The top 14 clusters of enriched terms originated from three GO annotations of biological processes, pathways retrieved from the KEGG database, and the Reactome Pathway Database. The enriched differentially expressed genes were associated with the terms “chromatin modifying enzymes” (R-HSA-3247509), “mRNA metabolic process” (GO: 0016071), “nuclear-transcribed mRNA catabolic process” (GO: 0000184), and “positive regulation of DNA-binding transcription factor activity” (GO: 0051091) **(** **Figure 5A** **)**. The top GO biological processes are shown in **Figure 5B**. The results confirmed that ZSCAN18 was a TF that might be involved in intracellular molecular and metabolic processes.

To further access molecular processes and pathways differentially activated in BC, GSEA of the ZSCAN18-low expression phenotype was performed **(** **Table S9** **)**. GSEA identified significant differences (false discovery rate [FDR] q-value < 0.05) in MSigDB collection (c2.cp.kegg.v6.2.symbols.gmt). The most enriched tumor-associated processes associated with low expression of ZSCAN18 were identified based on the NES, which included the cell cycle (NES = 1.952; FDR q-value = 0.025, < 0.05) and glycolysis signaling pathway (NES = 1.966; FDR q-value = 0.034, < 0.05) **(** **Figures 5C, D** **)**. Meanwhile, qRT-PCR was performed to identify gene associated with the Wnt/β-catenin and glycolysis signaling pathways. The results confirmed that overexpression of ZSCAN18 inhibited mRNA expression of CTNNB1, BCL9, TSC1, and PFKP in TNBC (MDA-MB-231) cells (p < 0.05) **(** **Figures 5E–H** **)**. In summary, downregulation of ZSCAN18 might be involved in the cell cycle and the Wnt/β-catenin and glycolysis signaling pathways in BC through transcription regulation.

### Association of ZSCAN18 with infiltration of immune cells in BC

**Figure 6:**
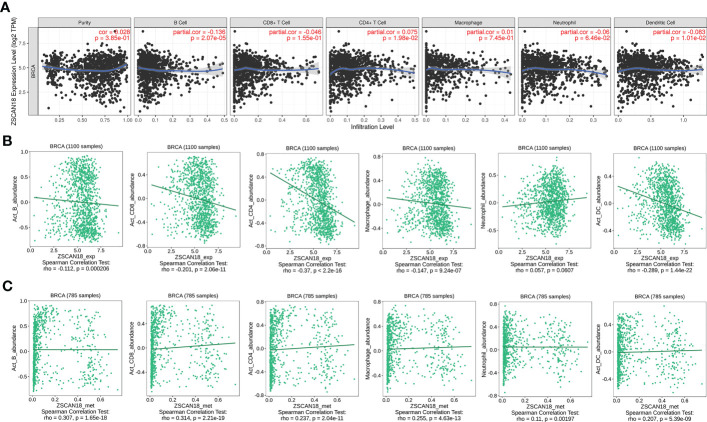
Correlation of ZSCAN18 expression/DNA methylation with immune infiltration in BC. **(A)** ZSCAN18 expression negatively correlated with infiltration levels of B cells and DCs in TIMER database. **(B)** ZSCAN18 expression showed negative association with infiltration levels of activated B cells, activated CD8 T cells, activated CD4 T cells, macrophages, and activated DCs in TISIDB database (n=1100). **(C)** ZSCAN18 DNA methylation positively associated with activated B cells, activated CD8 T cells, activated CD4 T cells, macrophages, neutrophils, and activated DCs in TISIDB database (n=785). The correlation coefficient (cor. and/or rho.) and p value were displayed.

The TIMER web server and the TISIDB were used to visualize the association of ZSCAN18 expression/methylation with the abundance of infiltrating immune cells in BC. The results revealed significant negative correlations between ZSCAN18 expression and TILs, including B cells (TIMER: p = 2.07E-05, < 0.001; TISIDB: p = 0.0002, < 0.001), activated CD8+ T cells (TISIDB: p = 2.06E-11, < 0.001), activated CD4+ T cells (TISIDB: p < 2.2E-16, < 0.001), macrophages (TISIDB: p = 9.24E-07, < 0.001), and DCs (TIMER: p = 1.01E-02, < 0.05; TISIDB: p = 1.44E-22, < 0.001) **(** **Figures 6A, B** **)**. Moreover, ZSCAN18 DNA methylation was positively associated with TILs, including activated B cells (p = 1.65E-18, < 0.001), activated CD8+ T cells (p = 2.21E-19, < 0.001), activated CD4+ T cells (p = 2.04E-11, < 0.001), macrophages (p = 4.63E-13, < 0.001), neutrophils (p = 0.00197, < 0.01), and activated DCs (p = 5.39E-09, < 0.001), as determined in reference to the TISIDB **(** **Figure 6C** **)**. These results confirmed the potential functions of ZSCAN18 expression/methylation in regulation of the immune microenvironment in BC. Correlation analysis revealed that ZSCAN18 expression/methylation was strongly associated with infiltration of immune cells in multiple human cancers **(** **Figures S3**, **S4** **)**.

### ZSCAN18-related hub genes and generation of a PPI network

**Figure 7:**
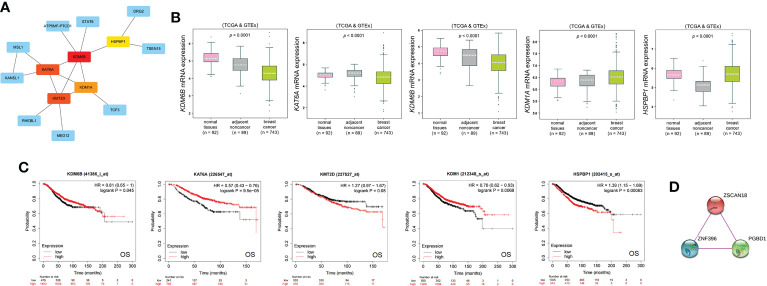
ZSCAN18-related hub genes and its PPI physical network. **(A)** Five ZSCAN18-related hub genes were identified by PPI network analysis using CytoHubba plugin of Cytoscape. The top five hub genes are shown in different colors except blue. **(B)** The mRNA expression of the top five hub genes in BC was observed based on bc-GenExMiner. The p value was displayed. **(C)** The correlation between the top five hub genes and OS of BC patients was investigated based on KM plotter. KDM1A: also called KDM1. The HR, 95% CI, and log-rank p were displayed. **(D)** A PPI physical network of ZSCAN18 was built by STRING database based on experimental evidence. The confidence score was defined as 0.7 in this analysis.

The top five ZSCAN18-related hub genes (KDM6B, KAT6A, KMT2D, KDM1A, and HSPBP1) identified by PPI network analysis based on the CytoHubba plugin of Cytoscape **(** **Figure 7A**; **Table S10** **)** were abnormally regulated in BC and associated with OS, as determined with the bc-GenExMiner mining tool and Kaplan–Meier plotter **(** **Figures 7B, C** **)**. A PPI physical subnetwork for ZSCAN18, based on experimental evidence, was generated using the STRING database of PPIs. The results showed that ZSCAN18, ZNF396, and PGBD1 were components of a physical complex **(** **Figure 7D** **)**.

## Discussion

In this study, the expression profile of ZSCAN18 in BC was evaluated. Bioinformatics analysis revealed significant downregulation of ZSCAN18 mRNA expression and decreased ZSCAN18 expression in the HER2-positive and TNBC subtypes. In addition, ZSCAN18 expression was relatively low in some BC cell lines. ZSCAN18 expression was negatively associated with HER2 status, the basal-like and TNBC subtypes, tumor stage, NPI, SBR grade, and mutated P53, and positively correlated with ER and PR status. The high extent of DNA methylation and low genetic alteration of ZSCAN18 in BC were identified as possible mechanisms underlying the regulation of ZSCAN18 expression. High expression of ZSCAN18 was correlated with better RFS, OS, and DMFS. These findings suggest that DNA methylation-regulated loss of ZSCAN18 expression may be a novel prognostic biomarker for BC.

To date, 54 members of the ZSCAN protein family have been identified. Although the structures are similar, the biological functions of ZSCAN proteins are diverse, especially in tumorigenesis and cancer progression (7). Our group previously reported that ZSCAN18 mRNA expression was downregulated in a variety of malignant tumors, including BC (**Figure 1A**), suggesting an important role as a tumor suppressor gene. However, relatively few studies have investigated the relevance of ZSCAN family proteins in human cancers, with the exception of the methylation status of ZSCAN18 in arterial diseases affecting the lower limbs (27) and metal concentrations in maternal blood samples (28). Most prior studies have focused on malignant tumors of the digestive tract, including cancers of the esophagus, stomach, pancreas, colon, rectum, bile duct, and liver, which found that ZSCAN18 methylation was common and negatively correlated with expression profiles, indicating the potential of ZSCAN18 methylation as a prognostic biomarker (8, 10–13, 29). A study of renal cell carcinoma found that ZSCAN18 knockdown promoted proliferation of human embryonic kidney cells and the demethylation drug azacitidine (Aza) reversed upregulation of ZSCAN18 protein expression in various renal cell carcinoma cell lines (9). In a recent research of gastric cancer, methylation-silenced ZSCAN18 induces the proliferation *via* weakening TP53INP2-mediated autophagy (14). Therefore, it is particularly important to explore the role of ZSCAN18 in BC from the perspective of epigenetics. In line with these previous studies, the results of the present study found that ZSCAN18 was downregulated in BC and DNA methylation was the possible mechanism underlying the regulation of ZSCAN18 expression. ZSCAN18 expression was also associated with survival of BC patients.

TFs control gene expression by recognizing specific DNA sequences. Families of TFs are classified based on conserved DNA-binding domains. The combination of chromatin immunoprecipitation (ChIP) assays with sequencing and other techniques have shown that TFs bind to the gene promoter in a specific environment and at a specific time to accurately regulate transcription (30). TFs can also act as promoters and/or suppressors of the expression of downstream target genes (31). To elucidate the molecular mechanism underlying the function of ZSCAN18 in BC, a ZSCAN18-associated regulatory network was constructed using multiple bioinformatics methods. First, the potential of ZSCAN18 as a TF in the regulation of downstream genes was investigated using GTRD analysis. The results showed that ZSCAN18 may be involved in intracellular molecular and metabolic processes. Subsequent GSEA showed that low ZSCAN18 expression was associated with the cell cycle and glycolysis signaling pathway, and possibly inhibition of genes related to the Wnt/β-catenin and glycolysis signaling pathways. Previous studies have reported that ZNF plays an important role in tumor aerobic glycolysis (Warburg effect) (32) and the Wnt/β-catenin signaling pathway (33, 34) through transcriptional regulation.

TILs, which comprise a mixture of T cells, B cells, macrophages, natural killer cells, neutrophils, and dendritic cells, have been observed in multiple human cancers, including BC. Hence, TILs may have prognostic value and could be related with improved responses to neoadjuvant therapies (35). However, it remains unclear whether ZSCAN18 is involved in the tumor immune microenvironment in BC. In this study, ZSCAN18 expression was negatively correlated with infiltration of B cells and DCs, as determined by the TIMER web server and reference to the TISIDB. However, the association of ZSCAN18 expression with other infiltrating TILs varied in BC, possibly because of differences in the number of TCGA samples and calculation methods. TIMER provides estimates of the abundance of TILs *via* multiple deconvolution-based analysis adjusted by tumor purity, thus the results are considered comparatively reliable. A recent study found a relationship between ZNF-based signatures and the tumor immune microenvironment in osteosarcoma (36). Emerging evidence has confirmed the value of DNA methylation to evaluate the immune response of tumors to improve diagnosis and treatment of breast and other cancers (37). Interestingly, in the TISIDB, ZSCAN18 DNA methylation is positively correlated with activated B cells, activated CD8+ and CD4+ T cells, macrophages, neutrophils, and activated DCs. Reportedly, site-specific CpG methylation is a useful marker to assess TILs and possibly to predict responses to checkpoint inhibitors for cancer treatment (38). The results of the present study highlight the potential of ZSCAN18 expression/DNA methylation in regulation of the tumor immune microenvironment in BC. The results also suggest that ZSCAN18 demethylation might be a valid strategy to improve the effects of immunotherapeutic agents. Thus, it is urgent to explore the mechanism and function of ZSCAN18 transcriptional expression/DNA methylation in regulation of the tumor microenvironment. Furthermore, Cytoscape and STRING analysis were used to identify the hub genes and physical complex of ZSCAN18. The results showed that five ZSCAN18-related hub genes (KDM6B, KAT6A, KMT2D, KDM1A, and HSPBP1) were abnormally regulated in BC and associated with OS. A recent research has reported that KDM6B can inhibit BC metastasis by regulating Wnt/β-catenin signaling (39), and another study has found PI3K pathway regulates ER-dependent transcription through KMT2D (40). KAT6A, a chromatin modifier, is considered as a candidate oncogene in luminal BC (41). Similarly, KDM1A inhibition is effective in reducing stemness and treating TNBC (42). Decreased HSPBP1 may lead to genomic instability and enables resistance to ionizing radiation in high-grade and metastatic BC (43). Therefore, these hub genes may associate with ZSCAN18 and play important roles in BC. In addition, ZSCAN18, ZNF396, and PGBD1 were identified as components of a physical complex. However, further studies are needed to evaluate the roles of these hub genes and complex.

In summary, ZSCAN18 is a potential prognostic and methylation-regulated tumor biomarker in BC. However, there were some limitations to this study that should be addressed. First, the analysis in this study mainly originated from public omics datasets, which should confirmed at the mRNA and protein levels. Second, some bioinformatics tools, such as the Kaplan–Meier plotter, have limited functionality without multivariable Cox regression analysis. Finally, direct evidence is lacking to verify the molecular mechanism of ZSCAN18 in BC. Hence, additional studies are urgently needed to validate the findings of this study of the role of ZSCAN18 as a tumor suppressor in BC.

## Data availability statement

The datasets presented in this study can be found in online repositories. The names of the repository/repositories and accession number(s) can be found in the article/**Supplementary Material**.

## Ethics statement

The informed consent from patients was waived because of the public nature of the database. Ethics approval was not required for this study.

## Author contributions

Study design: YF; investigation and resources: LH and YF; data collection: YW and YL; writing-original draft preparation: YW; writing-review and editing: SF and DF; data interpretation and visualization: YW and GP; supervision: QW, and YF; project administration: YL and YF; funding acquisition: YL and YF; statistical analysis: YW and YF. All authors contributed to the article and approved the submitted version.

## References

[1] Sung H Ferlay J Siegel RL Laversanne M Soerjomataram I Jemal A. Global cancer statistics 2020: GLOBOCAN estimates of incidence and mortality worldwide for 36 cancers in 185 countries. CA: Cancer J Clin (2021) 71(3):209–49. doi: 10.3322/caac.21660 33538338

[2] Chen W Zheng R Baade PD Zhang S Zeng H Bray F. Cancer statistics in China, 2015. CA: Cancer J Clin (2016) 66(2):115–32. doi: 10.3322/caac.21338 26808342

[3] Xiang TX Yuan Y Li LL Wang ZH Dan LY Chen Y. Aberrant promoter CpG methylation and its translational applications in breast cancer. Chin J Canc (2013) 32(1):12–20. doi: 10.5732/cjc.011.10344 PMC3845590 22059908

[4] Krishna SS Majumdar I Grishin NV. Structural classification of zinc fingers: survey and summary. Nucleic Acids Res (2003) 31(2):532–50. doi: 10.1093/nar/gkg161 PMC140525 12527760

[5] Edelstein LC Collins T. The SCAN domain family of zinc finger transcription factors. Gene. (2005) 359:1–17. doi: 10.1016/j.gene.2005.06.022 16139965

[6] Williams AJ Khachigian LM Shows T Collins T. Isolation and characterization of a novel zinc-finger protein with transcription repressor activity. J Biol Chem (1995) 270(38):22143–52. doi: 10.1074/jbc.270.38.22143 7673192

[7] Huang M Chen Y Han D Lei Z Chu X. Role of the zinc finger and SCAN domain-containing transcription factors in cancer. Am J Cancer Res (2019) 9(5):816–36. PMC6556609 31218096

[8] Vedeld HM Andresen K Eilertsen IA Nesbakken A Seruca R Gladhaug IP. The novel colorectal cancer biomarkers CDO1, ZSCAN18 and ZNF331 are frequently methylated across gastrointestinal cancers. Int J Canc (2015) 136(4):844–53. doi: 10.1002/ijc.29039 PMC4277335 24948044

[9] Morris MR Ricketts CJ Gentle D McRonald F Carli N Khalili H. Genome-wide methylation analysis identifies epigenetically inactivated candidate tumour suppressor genes in renal cell carcinoma. Oncogene (2011) 30(12):1390–401. doi: 10.1038/onc.2010.525 21132003

[10] Oka D Yamashita S Tomioka T Nakanishi Y Kato H Kaminishi M. The presence of aberrant DNA methylation in noncancerous esophageal mucosae in association with smoking history: a target for risk diagnosis and prevention of esophageal cancers. Cancer (2009) 115(15):3412–26. doi: 10.1002/cncr.24394 19472401

[11] Andresen K Boberg KM Vedeld HM Honne H Hektoen M Wadsworth CA. Novel target genes and a valid biomarker panel identified for cholangiocarcinoma. Epigenetics. (2012) 7(11):1249–57. doi: 10.4161/epi.22191 PMC3499326 22983262

[12] Mitchell SM Ross JP Drew HR Ho T Brown GS Saunders NF. A panel of genes methylated with high frequency in colorectal cancer. BMC canc (2014) 14:54. doi: 10.1186/1471-2407-14-54 PMC3924905 24485021

[13] Yu CB Zhu LY Wang YG Li F Zhang XY Dai WJ. Systemic transcriptome analysis of hepatocellular carcinoma. Tumour Biol J Int Soc Oncodevelopmental Biol Med (2016) 37(10):13323–31. doi: 10.1007/s13277-016-5286-5 27460080

[14] Li B Ren B Ma G Cai F Wang P Zeng Y. Inactivation of ZSCAN18 by promoter hypermethylation drives the proliferation *via* attenuating TP53INP2-mediated autophagy in gastric cancer cells. Clin Epigenetics (2023) 15(1):10. doi: 10.1186/s13148-023-01425-9 36650573 PMC9847086

[15] Li T Fan J Wang B Traugh N Chen Q Liu JS. TIMER: a web server for comprehensive analysis of tumor-infiltrating immune cells. Cancer Res (2017) 77(21):e108–e10. doi: 10.1158/0008-5472.CAN-17-0307 PMC6042652 29092952

[16] Chandrashekar DS Karthikeyan SK Korla PK Patel H Shovon AR Athar M. UALCAN: an update to the integrated cancer data analysis platform. Neoplasia. (2022) 25:18–27. doi: 10.1016/j.neo.2022.01.001 35078134 PMC8788199

[17] Jezequel P Gouraud W Ben Azzouz F Guerin-Charbonnel C Juin PP Lasla H. Bc-GenExMiner 4.5: new mining module computes breast cancer differential gene expression analyses. Database J Biol Database Curation (2021) 2021:baab007. doi: 10.1093/database/baab007 PMC7904047 33599248

[18] Nusinow DP Szpyt J Ghandi M Rose CM McDonald ER 3rd Kalocsay M. Quantitative proteomics of the cancer cell line encyclopedia. Cell. (2020) 180(2):387–402.e16. doi: 10.1016/j.cell.2019.12.023 31978347 PMC7339254

[19] Cerami E Gao J Dogrusoz U Gross BE Sumer SO Aksoy BA. The cBio cancer genomics portal: an open platform for exploring multidimensional cancer genomics data. Cancer discovery (2012) 2(5):401–4. doi: 10.1158/2159-8290.CD-12-0095 PMC3956037 22588877

[20] Madeira F Pearce M Tivey ARN Basutkar P Lee J Edbali O. Search and sequence analysis tools services from EMBL-EBI in 2022. Nucleic Acids Res (2022) 50(W1):W276–9. doi: 10.1093/nar/gkac240 PMC9252731 35412617

[21] Koch A Jeschke J Van Criekinge W van Engeland M De Meyer T. MEXPRESS update 2019. Nucleic Acids Res (2019) 47(W1):W561–W5. doi: 10.1093/nar/gkz445 PMC6602516 31114869

[22] Gyorffy B. Survival analysis across the entire transcriptome identifies biomarkers with the highest prognostic power in breast cancer. Comput Struct Biotechnol J (2021) 19:4101–9. doi: 10.1016/j.csbj.2021.07.014 PMC8339292 34527184

[23] Kolmykov S Yevshin I Kulyashov M Sharipov R Kondrakhin Y Makeev VJ. GTRD: an integrated view of transcription regulation. Nucleic Acids Res (2021) 49(D1):D104–D11. doi: 10.1093/nar/gkaa1057 PMC7778956 33231677

[24] Zhou Y Zhou B Pache L Chang M Khodabakhshi AH Tanaseichuk O. Metascape provides a biologist-oriented resource for the analysis of systems-level datasets. Nat Commun (2019) 10(1):1523. doi: 10.1038/s41467-019-09234-6 30944313 PMC6447622

[25] Subramanian A Tamayo P Mootha VK Mukherjee S Ebert BL Gillette MA. Gene set enrichment analysis: a knowledge-based approach for interpreting genome-wide expression profiles. Proc Natl Acad Sci U S A (2005) 102(43):15545–50. doi: 10.1073/pnas.0506580102 PMC1239896 16199517

[26] Ru B Wong CN Tong Y Zhong JY Zhong SSW Wu WC. TISIDB: an integrated repository portal for tumor-immune system interactions. Bioinf (Oxford England) (2019) 35(20):4200–2. doi: 10.1093/bioinformatics/btz210 30903160

[27] Bogucka-Kocka A Zalewski DP Ruszel KP Stepniewski A Galkowski D Bogucki J. Dysregulation of MicroRNA regulatory network in lower extremities arterial disease. Front Genet (2019) 10:1200. doi: 10.3389/fgene.2019.01200 31827490 PMC6892359

[28] Aung MT MB K Feinberg JI F Dou J DM J Mukherjee B. Maternal blood metal concentrations and whole blood DNA methylation during pregnancy in the early autism risk longitudinal investigation (EARLI). Epigenetics. (2022) 17(3):253–68. doi: 10.1080/15592294.2021.1897059 PMC8920182 33794742

[29] Andresen K Boberg KM Vedeld HM Honne H Jebsen P Hektoen M. Four DNA methylation biomarkers in biliary brush samples accurately identify the presence of cholangiocarcinoma. Hepatology. (2015) 61(5):1651–9. doi: 10.1002/hep.27707 PMC4832263 25644509

[30] Francois M Donovan P Fontaine F. Modulating transcription factor activity: interfering with protein-protein interaction networks. Semin Cell Dev Biol (2020) 99:12–9. doi: 10.1016/j.semcdb.2018.07.019 30172762

[31] Jolma A Yin Y Nitta KR Dave K Popov A Taipale M. DNA-Dependent formation of transcription factor pairs alters their binding specificity. Nature. (2015) 527(7578):384–8. doi: 10.1038/nature15518 26550823

[32] Zhou Y Lin F Wan T Chen A Wang H Jiang B. ZEB1 enhances warburg effect to facilitate tumorigenesis and metastasis of HCC by transcriptionally activating PFKM. Theranostics. (2021) 11(12):5926–38. doi: 10.7150/thno.56490 PMC8058737 33897890

[33] Liu G Jiang S Wang C Jiang W Liu Z Liu C. Zinc finger transcription factor 191, directly binding to beta-catenin promoter, promotes cell proliferation of hepatocellular carcinoma. Hepatology. (2012) 55(6):1830–9. doi: 10.1002/hep.25564 22213192

[34] Zhang C Xiang T Li S Ye L Feng Y Pei L. The novel 19q13 KRAB zinc-finger tumour suppressor ZNF382 is frequently methylated in oesophageal squamous cell carcinoma and antagonises wnt/beta-catenin signalling. Cell Death disease (2018) 9(5):573. doi: 10.1038/s41419-017-0087-3 29760376 PMC5951945

[35] Wein L Savas P Luen SJ Virassamy B Salgado R Loi S. Clinical validity and utility of tumor-infiltrating lymphocytes in routine clinical practice for breast cancer patients: current and future directions. Front Oncol (2017) 7:156. doi: 10.3389/fonc.2017.00156 28824872 PMC5540942

[36] Sun X Zheng D Guo W. Comprehensive analysis of a zinc finger protein gene-based signature with regard to prognosis and tumor immune microenvironment in osteosarcoma. Front Genet (2022) 13:835014. doi: 10.3389/fgene.2022.835014 35281811 PMC8914066

[37] Jeschke J Bizet M Desmedt C Calonne E Dedeurwaerder S Garaud S. DNA Methylation-based immune response signature improves patient diagnosis in multiple cancers. J Clin Invest (2017) 127(8):3090–102. doi: 10.1172/JCI91095 PMC5531413 28714863

[38] Bacolod MD Barany F Fisher PB. Can CpG methylation serve as surrogate markers for immune infiltration in cancer? Adv Cancer Res (2019) 143:351–84. doi: 10.1016/bs.acr.2019.03.007 31202362

[39] Xun J Gao R Wang B Li Y Ma Y Guan J. Histone demethylase KDM6B inhibits breast cancer metastasis by regulating wnt/beta-catenin signaling. FEBS Open bio (2021) 11(8):2273–81. doi: 10.1002/2211-5463.13236 PMC8329947 34165914

[40] Toska E Osmanbeyoglu HU Castel P Chan C Hendrickson RC Elkabets M. PI3K pathway regulates ER-dependent transcription in breast cancer through the epigenetic regulator KMT2D. Sci (New York NY) (2017) 355(6331):1324–30. doi: 10.1126/science.aah6893 PMC5485411 28336670

[41] Turner-Ivey B Guest ST Irish JC Kappler CS Garrett-Mayer E Wilson RC. KAT6A, a chromatin modifier from the 8p11-p12 amplicon is a candidate oncogene in luminal breast cancer. Neoplasia. (2014) 16(8):644–55. doi: 10.1016/j.neo.2014.07.007 PMC4234874 25220592

[42] Zhou M Venkata PP Viswanadhapalli S Palacios B Alejo S Chen Y. KDM1A inhibition is effective in reducing stemness and treating triple negative breast cancer. Breast Cancer Res Treat (2021) 185(2):343–57. doi: 10.1007/s10549-020-05963-1 33057995

[43] Youn CK Lee JH Hariharasudhan G Kim HB Kim J Lee S. HspBP1 is a dual function regulatory protein that controls both DNA repair and apoptosis in breast cancer cells. Cell Death Dis (2022) 13(4):309. doi: 10.1038/s41419-022-04766-0 35387978 PMC8986865

