# Supplementary material for: Zinc finger and SCAN domain-containing protein 18 is a potential DNA methylation-modified tumor suppressor and biomarker in breast cancer
Source: Front Endocrinol (Lausanne). 2023 May 8;14:1095604. doi: 10.3389/fendo.2023.1095604 (PMC10200902; doi:10.3389/fendo.2023.1095604)
Supplement: Supplementary file 1 [file DataSheet_1.zip › Supplementary Material/Figure S1.pdf]

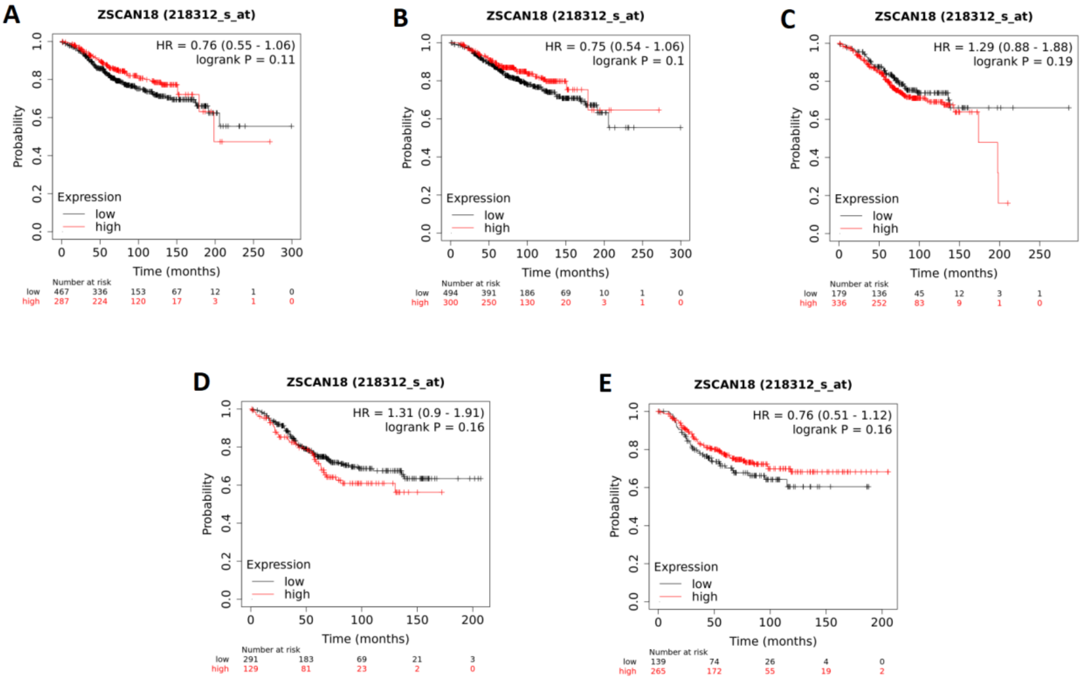

Figure S1 The prognostic value of ZSCAN18 in different molecular subtypes of breast cancer. No remarkable difference of OS was found in the groups of ER positive **(A)**, luminal A **(B)**, luminal B **(C)**, HER2 positive **(D)**, and basal-like **(E)** breast cancer. The hazards ratio (HR), 95% confidence interval (CI), and log-rank p were displayed.
