# Supplementary material for: Zinc finger and SCAN domain-containing protein 18 is a potential DNA methylation-modified tumor suppressor and biomarker in breast cancer
Source: Front Endocrinol (Lausanne). 2023 May 8;14:1095604. doi: 10.3389/fendo.2023.1095604 (PMC10200902; doi:10.3389/fendo.2023.1095604)
Supplement: Supplementary file 1 [file DataSheet_1.zip › Supplementary Material/Figure S2.pdf]

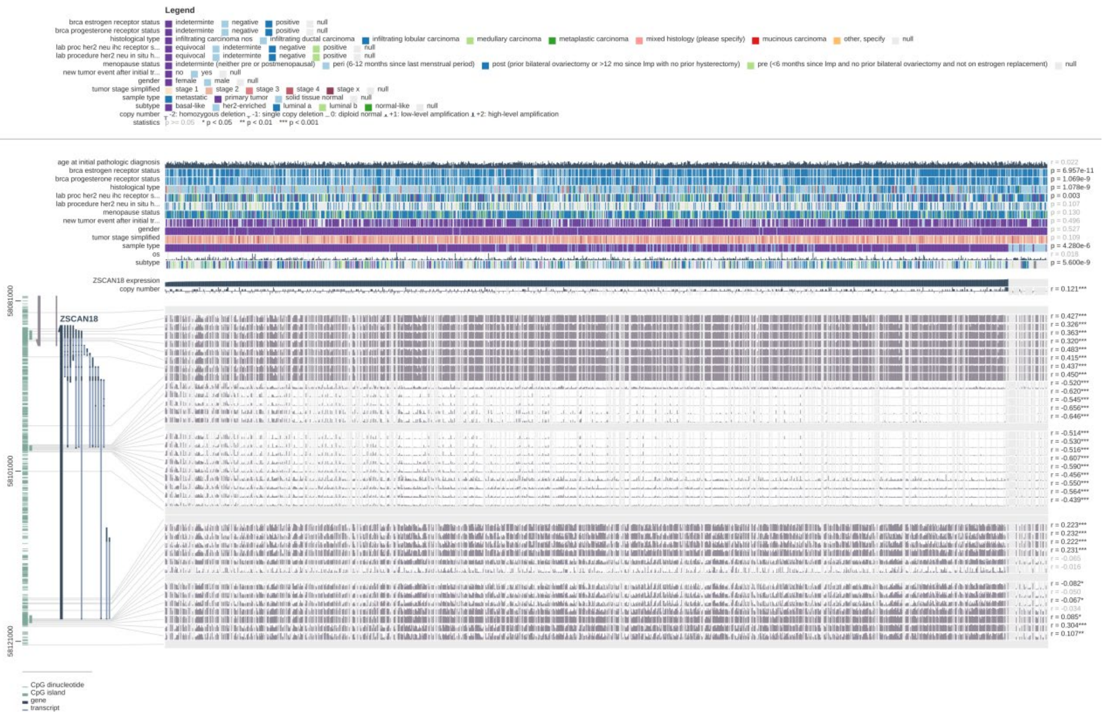

Figure S2 Visualization of DNA methylation and expression data of ZSCAN18 in breast invasive carcinoma analyzed by MEXPRESS. 1268 breast invasive carcinoma samples were included. DNA methylation data for each probe were determined using Infinium Human Methylation 450 microarray. Gene expression data were derived from Illumina HiSeq RNASeqV2. The samples are ascendingly ordered by the level of expression. The significance of the relation (correlation coefficient r or p value) between each row of data (clinical data, expression or methylation) was shown in the right side. *p<0.05; **p<0.01; ***p<0.001.
