# Supplementary material for: Zinc finger and SCAN domain-containing protein 18 is a potential DNA methylation-modified tumor suppressor and biomarker in breast cancer
Source: Front Endocrinol (Lausanne). 2023 May 8;14:1095604. doi: 10.3389/fendo.2023.1095604 (PMC10200902; doi:10.3389/fendo.2023.1095604)
Supplement: Supplementary file 1 [file DataSheet_1.zip › Supplementary Material/Figure S4.pdf]

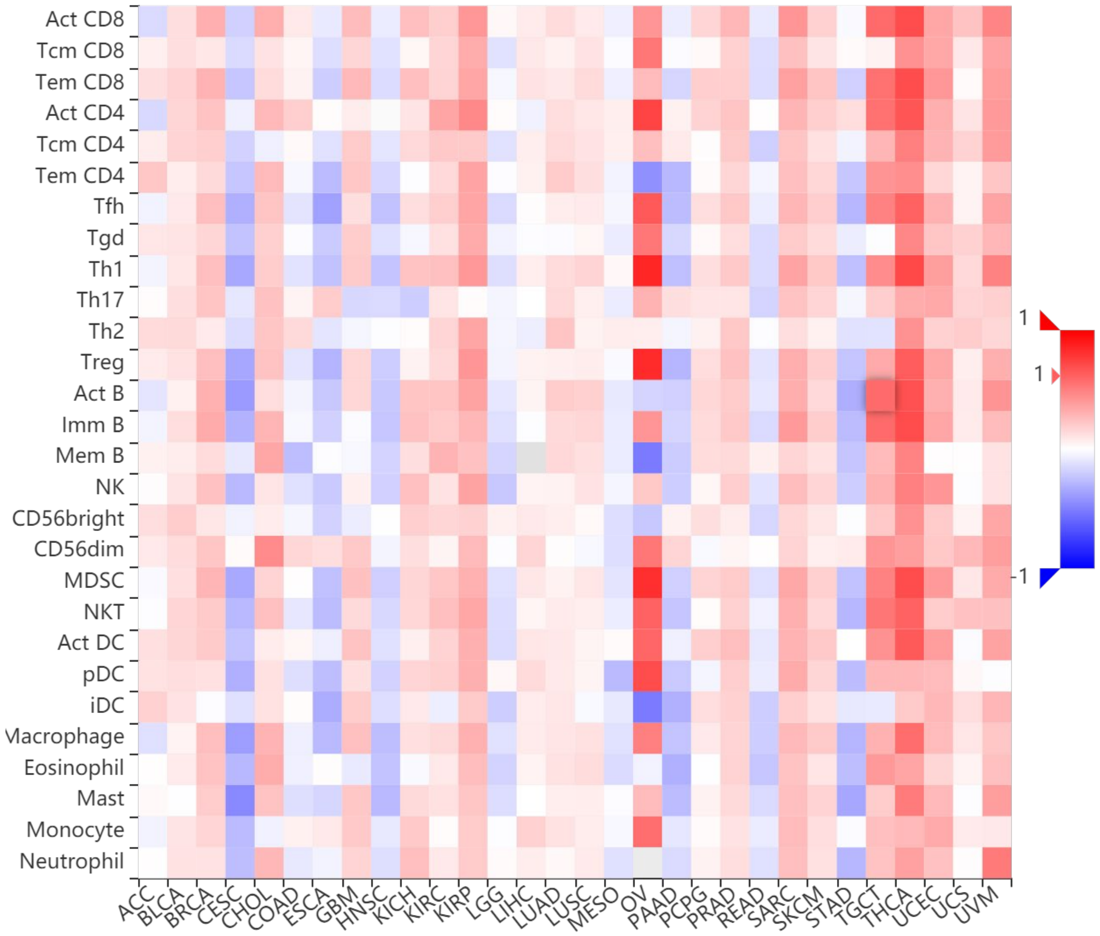

Figure S4 The correlation of ZSCAN18 DNA methylation with 28 types of TILs from multiple human cancers in TISIDB database. The gradation of color indicated the correlation coefficient.
