# Supplementary material for: Zinc finger and SCAN domain-containing protein 18 is a potential DNA methylation-modified tumor suppressor and biomarker in breast cancer
Source: Front Endocrinol (Lausanne). 2023 May 8;14:1095604. doi: 10.3389/fendo.2023.1095604 (PMC10200902; doi:10.3389/fendo.2023.1095604)
Supplement: Supplementary file 1 [file DataSheet_1.zip › Supplementary Material/Table S1.DOCX]

| **Name** | **Sequence（5’-3’）** | **Size** |
| --- | --- | --- |
| β-actin-F | CACCATTGGCAATGAGCGGTTC | 135bp |
| β-actin-R | AGGTCTTTGCGGATGTCCACGT |  |
| LDHA-F | GGATCTCCAACATGGCAGCCTT | 132bp |
| LDHA-R | AGACGGCTTTCTCCCTCTTGCT |  |
| PFKP-F | AGGCAGTCATCGCCTTGCTAGA | 130bp |
| PFKP-R | ATCGCCTTCTGCACATCCTGAG |  |
| BCL9-F | TCCAGCTCGTTCTCCCAACTTG | 152bp |
| BCL9-R | GATTGGAGTGAGAAAGTGGCTGG |  |
| CTNNB1-F | CACAAGCAGAGTGCTGAAGGTG | 146bp |
| CTNNB1-R | GATTCCTGAGAGTCCAAAGACAG |  |
| TSC-F | CTGGACAGACTGATACAGCAGG | 124bp |
| TSC-R | TGCGGATCTCATCTGAAGGAGG |  |

**Table S1 The sequences of primers.**
