# Supplementary material for: Zinc finger and SCAN domain-containing protein 18 is a potential DNA methylation-modified tumor suppressor and biomarker in breast cancer
Source: Front Endocrinol (Lausanne). 2023 May 8;14:1095604. doi: 10.3389/fendo.2023.1095604 (PMC10200902; doi:10.3389/fendo.2023.1095604)
Supplement: Supplementary file 1 [file DataSheet_1.zip › Supplementary Material/Table S10.DOCX]

**Table S10 The top five hub genes in the PPI network ranked by degree method**

| **Gene symbol** | **Gene description** | **Degree** |
| --- | --- | --- |
| KDM6B | lysine demethylase 6B | 6 |
| KAT6A | lysine acetyltransferase 6A | 5 |
| KMT2D | lysine methyltransferase 2D | 5 |
| KDM1A | lysine demethylase 1A | 4 |
| HSPBP1 | HSPA (Hsp70) binding protein 1 | 3 |
