# Supplementary material for: Zinc finger and SCAN domain-containing protein 18 is a potential DNA methylation-modified tumor suppressor and biomarker in breast cancer
Source: Front Endocrinol (Lausanne). 2023 May 8;14:1095604. doi: 10.3389/fendo.2023.1095604 (PMC10200902; doi:10.3389/fendo.2023.1095604)
Supplement: Supplementary file 1 [file DataSheet_1.zip › Supplementary Material/Table S2.DOCX]

| **Types of cancer** | **Types of normal tissues**  **(including tumor and metastasis)** | **p-value** |
| --- | --- | --- |
| BLCA.Tumor | BLCA.Normal | *1.64E-04* |
| BRCA.Tumor | BRCA.Normal | *2.43E-04* |
| CHOL.Tumor | CHOL.Normal | *1.40E-03* |
| COAD.Tumor | COAD.Normal | *1.64E-13* |
| ESCA.Tumor | ESCA.Normal | *9.03E-04* |
| HNSC-HPVpos.Tumor | HNSC-HPVneg.Tumor | 0.102 |
| HNSC.Tumor | HNSC.Normal | *4.68E-10* |
| KICH.Tumor | KICH.Normal | *4.98E-06* |
| KIRC.Tumor | KIRC.Normal | *2.44E-12* |
| KIRP.Tumor | KIRP.Normal | *1.12E-05* |
| LIHC.Tumor | LIHC.Normal | 0.187 |
| LUAD.Tumor | LUAD.Normal | *1.38E-07* |
| LUSC.Tumor | LUSC.Normal | *5.20E-11* |
| PRAD.Tumor | PRAD.Normal | *1.33E-06* |
| READ.Tumor | READ.Normal | *8.60E-05* |
| SKCM.Tumor | SKCM.Metastasis | *5.83E-03* |
| STAD.Tumor | STAD.Normal | *8.11E-06* |
| THCA.Tumor | THCA.Normal | *1.12E-05* |
| UCEC.Tumor | UCEC.Normal | *3.66E-19* |

**Table S2 The significant changes of ZSCAN18 mRNA in several human cancers** **constructed by TIMER based on TCGA datasets.**

**Note:** TIMER, tumor immune estimation resource; BLCA, bladder urothelial carcinoma; BRCA, breast invasive carcinoma; CHOL, cholangiocarcinoma; COAD, colon adenocarcinoma; ESCA, esophageal carcinoma; HNSC, head and neck squamous cell carcinoma; HPVpos, human papillomavirus positve; KICH, kidney chromophobe; KIRC, kidney renal clear cell carcinoma; KIRP, kidney renal papillary cell carcinoma; LIHC, liver hepatocellular carcinoma; LUAD, lung adenocarcinoma; LUSC, lung squamous cell carcinoma; PRAD, prostate adenocarcinoma; READ, rectum adenocarcinoma; SKCM, skin cutaneous melanoma; STAD, stomach adenocarcinoma; THCA, thyroid carcinoma; UCEC, uterine corpus endometrial carcinoma.
