# Supplementary material for: Zinc finger and SCAN domain-containing protein 18 is a potential DNA methylation-modified tumor suppressor and biomarker in breast cancer
Source: Front Endocrinol (Lausanne). 2023 May 8;14:1095604. doi: 10.3389/fendo.2023.1095604 (PMC10200902; doi:10.3389/fendo.2023.1095604)
Supplement: Supplementary file 1 [file DataSheet_1.zip › Supplementary Material/Table S4.DOCX]

| **Cell line** | **3 lineage** | **ZSCAN18 log2(TPM+1) Expression 22Q1 Public** |
| --- | --- | --- |
| DU4475 | ERneg HER2neg | 0 |
| SUM185PE | ERneg HER2neg | 0 |
| SUM52PE | ERpos HER2pos | 0 |
| MDAMB453 | ERneg HER2neg | 0.070389328 |
| HCC1569 | ERneg HER2pos | 0.070389328 |
| SUM159PT | ERneg HER2neg | 0.097610797 |
| HMC18 | ERneg HER2neg | 0.189033824 |
| SKBR3 | ERneg HER2pos | 0.201633861 |
| KPL1 | ERpos HER2neg | 0.23878686 |
| MCF7 | ERpos HER2neg | 0.432959407 |
| SUM229PE | ERneg HER2neg | 0.545968369 |
| SUM102PT | ERneg HER2neg | 0.575312331 |
| MDAMB415 | ERpos HER2neg | 1.925999419 |
| HS578T | ERneg HER2neg | 2.084064265 |
| SUM44PE | ERpos HER2pos | 4.011674533 |
| CAL851 | ERneg HER2neg | 4.115199749 |
| CAMA1 | ERpos HER2neg | 4.116031993 |
| MDAMB361 | ERpos HER2pos | 4.495695163 |
| MDAMB436 | ERneg HER2neg | 4.765534746 |
| MDAMB231 | ERneg HER2neg | 5.012568674 |
| HCC1428 | ERpos HER2neg | 5.043519494 |
| CAL51 | ERneg HER2neg | 5.211790976 |
| MDAMB468 | ERneg HER2neg | 6.11997861 |
| CAL120 | ERneg HER2neg | 6.749131973 |

**Table S4 The mRNA expression (log2 (TPM+1)) of ZSCAN18 in breast carcinoma cell lines constructed by CCLE.**

**Note:** CCLE, cancer database and the cell line encyclopedia; TPM, transcript per million; ERneg HER2neg, estrogen receptor negative and human epidermal growth factor receptor type 2 negative; ERpos HER2pos, estrogen receptor positive and human epidermal growth factor receptor type 2 positive; ERpos HER2neg, estrogen receptor positive and human epidermal growth factor receptor type 2 negative.
