# Supplementary material for: Zinc finger and SCAN domain-containing protein 18 is a potential DNA methylation-modified tumor suppressor and biomarker in breast cancer
Source: Front Endocrinol (Lausanne). 2023 May 8;14:1095604. doi: 10.3389/fendo.2023.1095604 (PMC10200902; doi:10.3389/fendo.2023.1095604)
Supplement: Supplementary file 1 [file DataSheet_1.zip › Supplementary Material/Table S5.DOCX]

**Table S5 The** **comparisons and** **statistical significance of ZSCAN18 protein expression in tumor subtypes of breast invasive carcinoma and normal control.**

| **Comparison** | **Statistical significance** |
| --- | --- |
| Normal vs. Luminal | *0.032* |
| Normal vs. HER2 Positive | *0.030* |
| Normal vs. TNBC | *1.35E-04* |
| Luminal vs. HER2 Positive | *6.27E-04* |
| Luminal vs. TNBC | *6.51E-08* |
| HER2 Positive vs. TNBC | 0.218 |

**Note:** HER2, human epidermal growth factor receptor type 2; TNBC, triple negative breast cancer.
