# Supplementary material for: Zinc finger and SCAN domain-containing protein 18 is a potential DNA methylation-modified tumor suppressor and biomarker in breast cancer
Source: Front Endocrinol (Lausanne). 2023 May 8;14:1095604. doi: 10.3389/fendo.2023.1095604 (PMC10200902; doi:10.3389/fendo.2023.1095604)
Supplement: Supplementary file 1 [file DataSheet_1.zip › Supplementary Material/Table S6.DOCX]

| **Comparison** | **Statistical significance** |
| --- | --- |
| Normal vs. Stage 1 | 0.889 |
| Normal vs. Stage 1 | *3.50E-03* |
| Normal vs. Stage 1 | 0.059 |
| Normal vs. Stage 1 | *0.018* |
| Stage 1 vs. Stage 2 | *0.042* |
| Stage 1 vs. Stage 3 | 0.126 |
| Stage 1 vs. Stage 4 | 0.058 |
| Stage 2 vs. Stage 3 | 0.593 |
| Stage 2 vs. Stage 4 | 0.274 |
| Stage 3 vs. Stage 4 | 0.147 |

**Table S6 The** **comparisons and** **statistical significance of ZSCAN18 transcript per million in tumor stage of breast invasive carcinoma and normal control.**
