# Supplementary material for: Zinc finger and SCAN domain-containing protein 18 is a potential DNA methylation-modified tumor suppressor and biomarker in breast cancer
Source: Front Endocrinol (Lausanne). 2023 May 8;14:1095604. doi: 10.3389/fendo.2023.1095604 (PMC10200902; doi:10.3389/fendo.2023.1095604)
Supplement: Supplementary file 1 [file DataSheet_1.zip › Supplementary Material/Table S7.DOCX]

| **Comparison** | **Statistical significance** |
| --- | --- |
| Normal vs. Luminal | *1.62E-12* |
| Normal vs. HER2 positive | *1.21E-04* |
| Normal vs. TNBC | *<1E-12* |
| Luminal vs. HER2 positive | 0.748 |
| Luminal vs. TNBC | *8.12E-05* |
| HER2 positive vs. TNBC | 0.228 |

**Table S7 The** **comparisons and** **statistical significance of ZSCAN18 promoter methylation level in tumor subtypes of breast invasive carcinoma and normal control.**

**Note:** HER2, human epidermal growth factor receptor type 2; TNBC, triple negative breast cancer.
