# Supplementary material for: Zinc finger and SCAN domain-containing protein 18 is a potential DNA methylation-modified tumor suppressor and biomarker in breast cancer
Source: Front Endocrinol (Lausanne). 2023 May 8;14:1095604. doi: 10.3389/fendo.2023.1095604 (PMC10200902; doi:10.3389/fendo.2023.1095604)
Supplement: Supplementary file 1 [file DataSheet_1.zip › Supplementary Material/Table S8.DOCX]

**Table S8 The genes regulated by ZSCAN18 predicted through GTRD platform.**

| **ID** | **Gene symbol** | **Site count** |
| --- | --- | --- |
| ENSG00000004487 | KDM1A | 1 |
| ENSG00000004660 | CAMKK1 | 1 |
| ENSG00000005100 | DHX33 | 1 |
| ENSG00000048162 | NOP16 | 1 |
| ENSG00000071564 | TCF3 | 1 |
| ENSG00000072071 | ADGRL1 | 1 |
| ENSG00000072849 | DERL2 | 1 |
| ENSG00000083168 | KAT6A | 1 |
| ENSG00000084070 | SMAP2 | 1 |
| ENSG00000090661 | CERS4 | 1 |
| ENSG00000092607 | TBX15 | 1 |
| ENSG00000099991 | CABIN1 | 1 |
| ENSG00000100320 | RBFOX2 | 1 |
| ENSG00000101203 | COL20A1 | 1 |
| ENSG00000101204 | CHRNA4 | 1 |
| ENSG00000105063 | PPP6R1 | 1 |
| ENSG00000106246 | PTCD1 | 1 |
| ENSG00000108175 | ZMIZ1 | 1 |
| ENSG00000108349 | CASC3 | 1 |
| ENSG00000108591 | DRG2 | 1 |
| ENSG00000108604 | SMARCD2 | 1 |
| ENSG00000108669 | CYTH1 | 1 |
| ENSG00000113719 | ERGIC1 | 1 |
| ENSG00000116698 | SMG7 | 1 |
| ENSG00000120071 | KANSL1 | 1 |
| ENSG00000123415 | SMUG1 | 1 |
| ENSG00000126243 | LRFN3 | 1 |
| ENSG00000132510 | KDM6B | 1 |
| ENSG00000133265 | HSPBP1 | 1 |
| ENSG00000135823 | STX6 | 1 |
| ENSG00000137478 | FCHSD2 | 1 |
| ENSG00000138639 | ARHGAP24 | 1 |
| ENSG00000138780 | GSTCD | 1 |
| ENSG00000138785 | INTS12 | 1 |
| ENSG00000140836 | ZFHX3 | 1 |
| ENSG00000142871 | CYR61 | 1 |
| ENSG00000143033 | MTF2 | 1 |
| ENSG00000143256 | PFDN2 | 1 |
| ENSG00000146066 | HIGD2A | 1 |
| ENSG00000146373 | RNF217 | 1 |
| ENSG00000150991 | UBC | 1 |
| ENSG00000151304 | SRFBP1 | 1 |
| ENSG00000153904 | DDAH1 | 1 |
| ENSG00000155093 | PTPRN2 | 1 |
| ENSG00000155592 | ZKSCAN2 | 1 |
| ENSG00000157388 | CACNA1D | 1 |
| ENSG00000159388 | BTG2 | 1 |
| ENSG00000160014 | CALM3 | 1 |
| ENSG00000160767 | FAM189B | 1 |
| ENSG00000162755 | KLHDC9 | 1 |
| ENSG00000165288 | BRWD3 | 1 |
| ENSG00000165716 | FAM69B | 1 |
| ENSG00000165934 | CPSF2 | 1 |
| ENSG00000166734 | CASC4 | 1 |
| ENSG00000166888 | STAT6 | 1 |
| ENSG00000167548 | KMT2D | 1 |
| ENSG00000167550 | RHEBL1 | 1 |
| ENSG00000170667 | RASA4B | 1 |
| ENSG00000174469 | CNTNAP2 | 1 |
| ENSG00000175414 | ARL10 | 1 |
| ENSG00000175416 | CLTB | 1 |
| ENSG00000177628 | GBA | 1 |
| ENSG00000179604 | CDC42EP4 | 1 |
| ENSG00000181291 | TMEM132E | 1 |
| ENSG00000183648 | NDUFB1 | 1 |
| ENSG00000183914 | DNAH2 | 1 |
| ENSG00000184634 | MED12 | 1 |
| ENSG00000185736 | ADARB2 | 1 |
| ENSG00000187147 | RNF220 | 1 |
| ENSG00000187775 | DNAH17 | 1 |
| ENSG00000187954 | CYHR1 | 2 |
| ENSG00000188511 | C22orf34 | 1 |
| ENSG00000188895 | MSL1 | 1 |
| ENSG00000196338 | NLGN3 | 1 |
| ENSG00000196914 | ARHGEF12 | 1 |
| ENSG00000198860 | TSEN15 | 1 |
| ENSG00000222439 | RNU6-994P | 1 |
| ENSG00000225470 | JPX | 1 |
| ENSG00000226548 | AC016722.3 | 1 |
| ENSG00000227630 | LINC01132 | 1 |
| ENSG00000227868 | C1orf234 | 1 |
| ENSG00000228022 | HCG20 | 1 |
| ENSG00000228906 | RP13-216E22.4 | 1 |
| ENSG00000229388 | RP11-442N24__B.1 | 1 |
| ENSG00000230510 | PPP5D1 | 1 |
| ENSG00000232860 | SMG7-AS1 | 1 |
| ENSG00000233016 | SNHG7 | 1 |
| ENSG00000233791 | LINC01136 | 1 |
| ENSG00000235689 | AP000351.13 | 1 |
| ENSG00000236699 | ARHGEF38 | 1 |
| ENSG00000237417 | XRCC6P1 | 1 |
| ENSG00000239332 | LINC01119 | 1 |
| ENSG00000240553 | RP1-184J9.2 | 1 |
| ENSG00000241468 | ATP5J2 | 1 |
| ENSG00000248919 | ATP5J2-PTCD1 | 1 |
| ENSG00000261564 | RP11-616M22.10 | 1 |
| ENSG00000262099 | CTC-524C5.5 | 1 |
| ENSG00000263006 | ROCK1P1 | 1 |
| ENSG00000265345 | MIR5188 | 1 |
| ENSG00000267649 | CTD-2587H24.10 | 1 |
| ENSG00000267786 | AF038458.3 | 1 |
| ENSG00000268423 | AC011551.3 | 1 |
| ENSG00000270103 | RNU11 | 1 |
| ENSG00000271717 | CTD-3020H12.4 | 1 |
| ENSG00000272691 | RP11-290M5.4 | 1 |
| ENSG00000272886 | DCP1A | 1 |
| ENSG00000272949 | RP11-514P8.8 | 1 |
| ENSG00000274356 | RP11-216L13.21 | 1 |
| ENSG00000274430 | JPX_1 | 1 |
| ENSG00000274925 | CTD-2547G23.4 | 1 |
| ENSG00000274978 | RNU11 | 1 |
| ENSG00000274998 | SNORA17A | 1 |
| ENSG00000275198 | RP11-471B22.3 | 1 |
| ENSG00000275585 | CH17-118O6.3 | 1 |
| ENSG00000276161 | SNORA17B | 1 |
| ENSG00000276784 | JPX_2 | 1 |
| ENSG00000278860 | RP11-28A22.2 | 1 |
| ENSG00000280335 | RP11-15F12.6 | 1 |
| ENSG00000280496 | SNORA17B | 1 |
| ENSG00000281808 | SNORA17 | 1 |
| ENSG00000282057 | RP4-621F18.2 | 1 |
| ENSG00000282097 | RP4-781K5.7 | 1 |
