# Supplementary material for: Zinc finger and SCAN domain-containing protein 18 is a potential DNA methylation-modified tumor suppressor and biomarker in breast cancer
Source: Front Endocrinol (Lausanne). 2023 May 8;14:1095604. doi: 10.3389/fendo.2023.1095604 (PMC10200902; doi:10.3389/fendo.2023.1095604)
Supplement: Supplementary file 1 [file DataSheet_1.zip › Supplementary Material/Table S9.DOCX]

**Table S9 Gene sets enriched in phenotype low ZSCAN18 expression**

| **NAME** | **ES** | **NES** | **NOM p-val** | **FDR q-val** |
| --- | --- | --- | --- | --- |
| KEGG_PATHOGENIC_ESCHERICHIA_COLI_INFECTION | 0.632 | 2.176 | 0.002 | 0.013 |
| KEGG_PROTEASOME | 0.792 | 2.085 | 0.000 | 0.024 |
| KEGG_GLYCOSPHINGOLIPID_BIOSYNTHESIS_LACTO_AND_NEOLACTO_SERIES | 0.633 | 2.008 | 0.002 | 0.043 |
| KEGG_CYSTEINE_AND_METHIONINE_METABOLISM | 0.582 | 2.004 | 0.000 | 0.034 |
| KEGG_CITRATE_CYCLE_TCA_CYCLE | 0.702 | 1.987 | 0.002 | 0.032 |
| KEGG_GLYCOLYSIS_GLUCONEOGENESIS | 0.557 | 1.966 | 0.002 | 0.034 |
| KEGG_TERPENOID_BACKBONE_BIOSYNTHESIS | 0.758 | 1.966 | 0.002 | 0.029 |
| KEGG_ANTIGEN_PROCESSING_AND_PRESENTATION | 0.663 | 1.954 | 0.010 | 0.028 |
| KEGG_CELL_CYCLE | 0.548 | 1.952 | 0.012 | 0.025 |
| KEGG_NATURAL_KILLER_CELL_MEDIATED_CYTOTOXICITY | 0.557 | 1.908 | 0.012 | 0.035 |
| KEGG_STEROID_BIOSYNTHESIS | 0.723 | 1.884 | 0.010 | 0.040 |
| KEGG_AMINO_SUGAR_AND_NUCLEOTIDE_SUGAR_METABOLISM | 0.541 | 1.848 | 0.002 | 0.050 |
| KEGG_TOLL_LIKE_RECEPTOR_SIGNALING_PATHWAY | 0.511 | 1.834 | 0.012 | 0.052 |
| KEGG_PENTOSE_PHOSPHATE_PATHWAY | 0.595 | 1.786 | 0.008 | 0.071 |
| KEGG_OOCYTE_MEIOSIS | 0.444 | 1.781 | 0.017 | 0.069 |
| KEGG_DNA_REPLICATION | 0.673 | 1.775 | 0.039 | 0.068 |
| KEGG_TYPE_I_DIABETES_MELLITUS | 0.709 | 1.770 | 0.028 | 0.067 |
| KEGG_GRAFT_VERSUS_HOST_DISEASE | 0.778 | 1.770 | 0.030 | 0.063 |
| KEGG_PYRUVATE_METABOLISM | 0.523 | 1.765 | 0.008 | 0.061 |
| KEGG_NOD_LIKE_RECEPTOR_SIGNALING_PATHWAY | 0.516 | 1.755 | 0.014 | 0.063 |
| KEGG_PYRIMIDINE_METABOLISM | 0.467 | 1.744 | 0.021 | 0.066 |
| KEGG_RNA_POLYMERASE | 0.573 | 1.698 | 0.037 | 0.089 |
| KEGG_SYSTEMIC_LUPUS_ERYTHEMATOSUS | 0.630 | 1.690 | 0.045 | 0.089 |
| KEGG_FC_GAMMA_R_MEDIATED_PHAGOCYTOSIS | 0.440 | 1.675 | 0.030 | 0.094 |
| KEGG_VIRAL_MYOCARDITIS | 0.549 | 1.674 | 0.052 | 0.091 |
| KEGG_ARGININE_AND_PROLINE_METABOLISM | 0.478 | 1.655 | 0.012 | 0.099 |
| KEGG_GALACTOSE_METABOLISM | 0.526 | 1.641 | 0.031 | 0.106 |
| KEGG_P53_SIGNALING_PATHWAY | 0.417 | 1.612 | 0.029 | 0.122 |
| KEGG_AMYOTROPHIC_LATERAL_SCLEROSIS_ALS | 0.430 | 1.610 | 0.020 | 0.119 |
| KEGG_PURINE_METABOLISM | 0.380 | 1.608 | 0.018 | 0.116 |
| KEGG_LEISHMANIA_INFECTION | 0.542 | 1.594 | 0.074 | 0.122 |
| KEGG_ALANINE_ASPARTATE_AND_GLUTAMATE_METABOLISM | 0.471 | 1.591 | 0.037 | 0.121 |
| KEGG_REGULATION_OF_ACTIN_CYTOSKELETON | 0.382 | 1.585 | 0.036 | 0.121 |
| KEGG_AUTOIMMUNE_THYROID_DISEASE | 0.583 | 1.567 | 0.066 | 0.130 |
| KEGG_VIBRIO_CHOLERAE_INFECTION | 0.463 | 1.566 | 0.041 | 0.128 |
| KEGG_ALLOGRAFT_REJECTION | 0.713 | 1.565 | 0.083 | 0.125 |
| KEGG_HOMOLOGOUS_RECOMBINATION | 0.532 | 1.557 | 0.074 | 0.127 |
| KEGG_PRIMARY_IMMUNODEFICIENCY | 0.641 | 1.556 | 0.099 | 0.125 |
| KEGG_GLYCOSAMINOGLYCAN_BIOSYNTHESIS_KERATAN_SULFATE | 0.553 | 1.546 | 0.032 | 0.128 |
| KEGG_GLIOMA | 0.390 | 1.543 | 0.042 | 0.127 |
| KEGG_MISMATCH_REPAIR | 0.561 | 1.543 | 0.101 | 0.124 |
| KEGG_CELL_ADHESION_MOLECULES_CAMS | 0.453 | 1.526 | 0.107 | 0.133 |
| KEGG_GLYOXYLATE_AND_DICARBOXYLATE_METABOLISM | 0.553 | 1.525 | 0.066 | 0.130 |
| KEGG_PROTEIN_EXPORT | 0.560 | 1.518 | 0.079 | 0.131 |
| KEGG_CHEMOKINE_SIGNALING_PATHWAY | 0.409 | 1.512 | 0.078 | 0.133 |
| KEGG_ONE_CARBON_POOL_BY_FOLATE | 0.529 | 1.511 | 0.073 | 0.130 |
| KEGG_CYTOKINE_CYTOKINE_RECEPTOR_INTERACTION | 0.410 | 1.506 | 0.085 | 0.131 |
| KEGG_CYTOSOLIC_DNA_SENSING_PATHWAY | 0.441 | 1.506 | 0.060 | 0.128 |
| KEGG_PENTOSE_AND_GLUCURONATE_INTERCONVERSIONS | 0.536 | 1.502 | 0.054 | 0.129 |
| KEGG_ALZHEIMERS_DISEASE | 0.416 | 1.493 | 0.092 | 0.132 |
| KEGG_INTESTINAL_IMMUNE_NETWORK_FOR_IGA_PRODUCTION | 0.567 | 1.475 | 0.148 | 0.141 |
| KEGG_STARCH_AND_SUCROSE_METABOLISM | 0.429 | 1.431 | 0.067 | 0.170 |
| KEGG_BLADDER_CANCER | 0.392 | 1.429 | 0.082 | 0.169 |
| KEGG_PROGESTERONE_MEDIATED_OOCYTE_MATURATION | 0.363 | 1.416 | 0.075 | 0.176 |
| KEGG_LEUKOCYTE_TRANSENDOTHELIAL_MIGRATION | 0.380 | 1.414 | 0.101 | 0.174 |
| KEGG_TRYPTOPHAN_METABOLISM | 0.420 | 1.412 | 0.084 | 0.173 |
| KEGG_RNA_DEGRADATION | 0.400 | 1.400 | 0.120 | 0.180 |
| KEGG_DORSO_VENTRAL_AXIS_FORMATION | 0.450 | 1.386 | 0.111 | 0.190 |
| KEGG_RENAL_CELL_CARCINOMA | 0.356 | 1.385 | 0.114 | 0.188 |
| KEGG_ADHERENS_JUNCTION | 0.371 | 1.366 | 0.124 | 0.202 |
| KEGG_B_CELL_RECEPTOR_SIGNALING_PATHWAY | 0.403 | 1.346 | 0.188 | 0.217 |
| KEGG_PRION_DISEASES | 0.452 | 1.343 | 0.154 | 0.217 |
| KEGG_FRUCTOSE_AND_MANNOSE_METABOLISM | 0.409 | 1.342 | 0.139 | 0.214 |
| KEGG_DRUG_METABOLISM_OTHER_ENZYMES | 0.394 | 1.337 | 0.135 | 0.215 |
| KEGG_PANCREATIC_CANCER | 0.343 | 1.333 | 0.147 | 0.216 |
| KEGG_LYSOSOME | 0.373 | 1.332 | 0.166 | 0.213 |
| KEGG_PARKINSONS_DISEASE | 0.436 | 1.303 | 0.224 | 0.236 |
| KEGG_GAP_JUNCTION | 0.321 | 1.301 | 0.122 | 0.235 |
| KEGG_EPITHELIAL_CELL_SIGNALING_IN_HELICOBACTER_PYLORI_INFECTION | 0.353 | 1.295 | 0.140 | 0.238 |
| KEGG_PATHWAYS_IN_CANCER | 0.290 | 1.288 | 0.147 | 0.241 |
| KEGG_GLUTATHIONE_METABOLISM | 0.394 | 1.281 | 0.188 | 0.245 |
| KEGG_NICOTINATE_AND_NICOTINAMIDE_METABOLISM | 0.384 | 1.275 | 0.174 | 0.247 |
| KEGG_NON_SMALL_CELL_LUNG_CANCER | 0.342 | 1.264 | 0.179 | 0.255 |
| KEGG_BIOSYNTHESIS_OF_UNSATURATED_FATTY_ACIDS | 0.419 | 1.250 | 0.204 | 0.266 |
| KEGG_LONG_TERM_POTENTIATION | 0.312 | 1.233 | 0.197 | 0.280 |
| KEGG_T_CELL_RECEPTOR_SIGNALING_PATHWAY | 0.358 | 1.226 | 0.274 | 0.283 |
| KEGG_APOPTOSIS | 0.327 | 1.225 | 0.221 | 0.281 |
| KEGG_ERBB_SIGNALING_PATHWAY | 0.304 | 1.224 | 0.203 | 0.278 |
| KEGG_STEROID_HORMONE_BIOSYNTHESIS | 0.360 | 1.204 | 0.216 | 0.296 |
| KEGG_SMALL_CELL_LUNG_CANCER | 0.300 | 1.182 | 0.237 | 0.318 |
| KEGG_HUNTINGTONS_DISEASE | 0.330 | 1.175 | 0.297 | 0.321 |
| KEGG_OXIDATIVE_PHOSPHORYLATION | 0.407 | 1.174 | 0.318 | 0.318 |
| KEGG_AXON_GUIDANCE | 0.282 | 1.153 | 0.265 | 0.338 |
| KEGG_PHENYLALANINE_METABOLISM | 0.410 | 1.145 | 0.288 | 0.344 |
| KEGG_O_GLYCAN_BIOSYNTHESIS | 0.355 | 1.143 | 0.276 | 0.342 |
| KEGG_BASAL_TRANSCRIPTION_FACTORS | 0.349 | 1.141 | 0.309 | 0.341 |
| KEGG_PROSTATE_CANCER | 0.282 | 1.116 | 0.300 | 0.366 |
| KEGG_PORPHYRIN_AND_CHLOROPHYLL_METABOLISM | 0.356 | 1.110 | 0.321 | 0.369 |
| KEGG_FOCAL_ADHESION | 0.285 | 1.083 | 0.363 | 0.399 |
| KEGG_RIBOFLAVIN_METABOLISM | 0.368 | 1.081 | 0.345 | 0.397 |
| KEGG_ASTHMA | 0.441 | 1.080 | 0.423 | 0.393 |
| KEGG_N_GLYCAN_BIOSYNTHESIS | 0.312 | 1.079 | 0.341 | 0.391 |
| KEGG_JAK_STAT_SIGNALING_PATHWAY | 0.282 | 1.068 | 0.370 | 0.401 |
| KEGG_CARDIAC_MUSCLE_CONTRACTION | 0.333 | 1.058 | 0.382 | 0.409 |
| KEGG_THYROID_CANCER | 0.307 | 1.049 | 0.360 | 0.417 |
| KEGG_GLYCOSPHINGOLIPID_BIOSYNTHESIS_GANGLIO_SERIES | 0.372 | 1.044 | 0.406 | 0.419 |
| KEGG_GLYCOSAMINOGLYCAN_DEGRADATION | 0.352 | 1.013 | 0.440 | 0.454 |
| KEGG_RIG_I_LIKE_RECEPTOR_SIGNALING_PATHWAY | 0.272 | 1.005 | 0.453 | 0.459 |
| KEGG_PPAR_SIGNALING_PATHWAY | 0.298 | 1.000 | 0.436 | 0.460 |
| KEGG_MELANOMA | 0.249 | 0.997 | 0.441 | 0.459 |
| KEGG_HEMATOPOIETIC_CELL_LINEAGE | 0.327 | 0.987 | 0.475 | 0.469 |
| KEGG_BUTANOATE_METABOLISM | 0.303 | 0.986 | 0.460 | 0.466 |
| KEGG_BETA_ALANINE_METABOLISM | 0.308 | 0.985 | 0.449 | 0.463 |
| KEGG_PROPANOATE_METABOLISM | 0.310 | 0.980 | 0.462 | 0.465 |
| KEGG_SPLICEOSOME | 0.280 | 0.933 | 0.491 | 0.524 |
| KEGG_AMINOACYL_TRNA_BIOSYNTHESIS | 0.331 | 0.904 | 0.532 | 0.560 |
| KEGG_ASCORBATE_AND_ALDARATE_METABOLISM | 0.336 | 0.894 | 0.569 | 0.569 |
| KEGG_RENIN_ANGIOTENSIN_SYSTEM | 0.297 | 0.882 | 0.617 | 0.582 |
| KEGG_ECM_RECEPTOR_INTERACTION | 0.270 | 0.860 | 0.595 | 0.609 |
| KEGG_COMPLEMENT_AND_COAGULATION_CASCADES | 0.315 | 0.759 | 0.797 | 0.753 |
| KEGG_RIBOSOME | 0.257 | 0.595 | 0.764 | 0.926 |

NES: normalized enrichment score; NOM: nominal; FDR: false discovery rate. Gene sets with FDR q-val<0.05 are considered as significant.
